# Necroptosis and Prostate Cancer: Molecular Mechanisms and Therapeutic Potential

**DOI:** 10.3390/cells11071221

**Published:** 2022-04-04

**Authors:** Giovanni Luca Beretta, Nadia Zaffaroni

**Affiliations:** Molecular Pharmacology Unit, Department of Applied Research and Technological Development, Fondazione IRCCS Istituto Nazionale dei Tumori di Milano, 20133 Milan, Italy; giovanni.beretta@istitutotumori.mi.it

**Keywords:** necroptosis, prostate cancer, RIP1, RIP3, MLKL

## Abstract

Necroptosis is a programmed form of necrosis characterized by mitochondrial alterations and plasma membrane permeabilization resulting in the release of cytoplasmic content into extracellular space, and leading to inflammatory reactions. Besides its critical role in viral defense mechanisms and inflammatory diseases, necroptosis plays pivotal functions in the drug response of tumors, including prostate cancer. Necroptosis is mainly governed by kinase enzymes, including RIP1, RIP3, and MLKL, and conversely to apoptosis, is a caspase-independent mechanism of cell death. Numerous compounds induce necroptosis in prostate cancer models, including (i) compounds of natural origin, (ii) synthetic and semisynthetic small molecules, and (iii) selenium and selenium-based nanoparticles. Here, we overview the molecular mechanisms underlying necroptosis and discuss the possible implications of drugs inducing necroptosis for prostate cancer therapy.

## 1. Introduction

Cell death mechanisms include accidental cell death (ACD) and regulated cell death (RCD) [1]. External extreme temperature and pressure, chemical stress, and osmotic pressure exceeding the capability of the cell to restore a physiologic condition lead to ACD. This type of cell death is an uncontrolled process in which the hallmark is the cell membrane rupturing, causing the spillover of the cytoplasm into the extracellular environment. Alterations in the cell membrane and the release of cellular content are also typical features of RCD [1,2]. RCD is controlled by molecules of specific signal cascades governing biochemical, morphological, and immunological consequences in a regulated and programmed manner. RCD guides pivotal steps of physiological and pathological processes [1]. Besides the most studied apoptosis, RCD includes autophagy, pyroptosis, ferroptosis, and necroptosis (NEC) [3,4,5,6,7,8,9]. RCD is a concept in continuous evolution. Very recently, PANoptosis, which embraces pyroptosis, apoptosis, and NEC, has emerged as a new RCD mode controlled by the aggregation of specific effector molecules leading to the formation of the PANoptosome [10,11]. The role played by PANoptosis in tumors was reported in colorectal cancer [10].

Differences in cell morphology, gene expression, and biochemical properties classify diverse forms of RCD. Therefore, molecules governing different pathways of different RCDs can be considered to be biomarkers and potential therapeutic targets [12]. The pharmacological strategy to hit selective factors of specific RCD pathways showed efficacy for cancer therapy [13]. In this context, apoptosis is paradigmatic, and the development of drugs inducing apoptosis represented the major goal of medical research [14]. However, this strategy has demonstrated numerous limits, and the emergence of drug resistance rendering tumors insensitive to apoptosis is crucial [15]. In this scenario, the discovery of drugs that induce nonapoptotic RCD is an intriguing approach for anticancer therapy, and compounds inducing NEC in tumors, including prostate cancer (PCa), are interesting [16,17,18].

PCa is the second most diagnosed tumor (incidence of 14.1% over a total of 10.1 million new cases) and the fifth global cause of cancer death (mortality of 6.8% over a total of 5.5 million deaths) in men [19]. Men affected by localized or locally advanced PCa are treated by radical prostatectomy and radiotherapy. Conversely, metastatic androgen-sensitive PCa-suffering patients undergo androgen deprivation therapy (ADT). The disease invariably progresses towards lethal metastatic castration-resistant PCa (CRPC) that is insensitive or resistant to ADT. In spite of several efforts devised thus far in the development of new effective therapeutics on CRPC patients, including taxane-based chemotherapy (cabazitaxel and docetaxel), inhibitors of androgen synthesis (abiraterone) or signaling (enzalutamide), bone-targeting radiotherapy (radium-223) and immunotherapy (sipuleucel-T), the disease still remains lethal [20]. CRPC patients among all PCa patients, and their poor prognosis and survival reflect the need to develop other drugs. Thus, the discovery of new antitumor drugs and active medical interventions on CRPC patients is urgent, and compounds that induce NEC are expected to produce benefits [17,21].

Here, we overview the molecular mechanisms underlying NEC and discuss the possible implications of drug-inducing NEC for PCa therapy.

## 2. Apoptosis and Necroptosis: Overview on Molecular Mechanisms

In 2005, Degterev and coworkers described NEC as a peculiar type of necrosis controlled by a specific signaling cascade that is inhibited by necrostatin 1 (Nec1) [22]. Taking advantage of a siRNA-mediated approach, receptor-interacting serine/threonine protein kinase 1 (RIP1) was identified as the target of Nec1. In 2018, such a type of programmed necrosis, not dependent on the activation of cysteine-aspartic proteases (caspases), and mainly governed by RIP1, receptor-interacting serine/threonine protein kinase 3 (RIP3) and substrate mixed lineage kinase domain like pseudokinase (MLKL) was called NEC [23]. NEC controls cell homeostasis, neurodegeneration, infectious diseases, inflammation, cardiovascular and skin diseases, acute kidney injury, and cancer [24].

Cells undergoing NEC and necrosis show similar behavior [25,26]. Although trauma, toxic stress, and infection are responsible for both necrosis and NEC, the latter differs from necrosis as a finely controlled type of RCD. Specific signaling pathways, including TNRF1 and RIP, govern NEC induction via the formation of the necroptosome complex. No important differences in morphology characterize cells undergoing NEC and necrosis (e.g., organelle and cell swelling, loss of membrane integrity, and the release of intracellular content). Moreover, both cell death mechanisms induce mitochondrial dysfunction with mitochondrial membrane collapse. However, only during NEC the production of reactive oxygen species (ROS) and the release of apoptosis-inducing factor (AIF) occur. Proinflammatory response characterizes both NEC and necrosis. Nec1 counteracts only NEC, while it is ineffective on necrosis.

**Figure 1:**
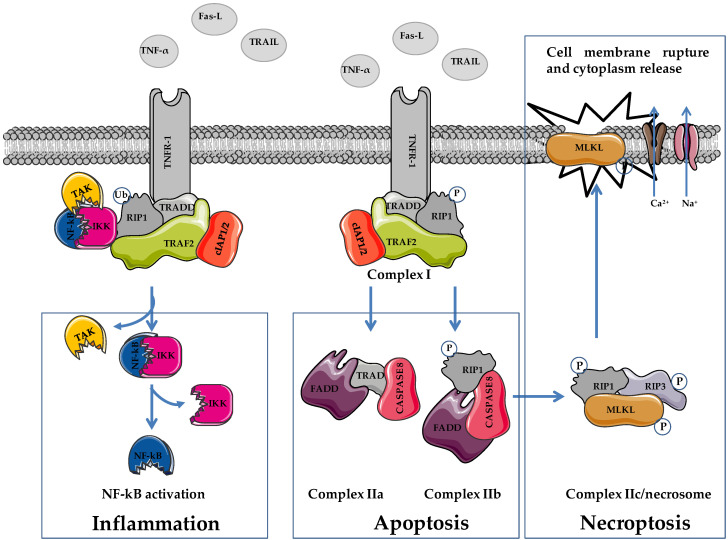
Schematic representation of cellular pathways involved in necroptosis. TNF-α, tumor necrosis factor α; FasL, Fas ligand; TRAIL, TNF-related apoptosis-inducing ligand; TNFR1, TNF receptor 1; RIP1/3, receptor-interacting serine/threonine-protein kinase 1/3; MLKL, mixed lineage linase domain like pseudokinase; TRADD, TNF receptor associated death domain protein; FADD, Fas-associated death domain; TRAF2, TNF receptor associated factor 2; cIAP1/2, cellular inhibitor of apoptosis proteins 1/2; TAK complex formed by TAK1, TAB2 and TAB3; IKK complex, formed by IKKα and IKKβ. Figure was prepared using tools from Servier Medical Art (http://www.servier.fr/servier-medical-art, accessed on 15 March 2022).

Tumor necrosis factor-α (TNF-α), Fas ligand (FasL), and TNF-related apoptosis-inducing ligand (TRAIL) are death-inducing molecules acting on FasL/TNF receptor (TNFR)1, which can induce either apoptosis or NEC depending on caspase functionality (Figure 1) [27,28].

Cells undergoing apoptosis arrest their growth and division, and activate plasma membrane modifications, chromatin condensation, and DNA fragmentation (DNA ladder), and cell shrinkage favoring the formation of apoptotic bodies engulfing the surrounding environmental phagocytes (Table 1). During NEC, a cell increases its volume, and shows organelle shrinkage and plasma membrane disintegration. Cellular contents, including damage-related pattern molecules (DAMPs), high-mobility group box 1 (HMGB1), and mitochondrial DNA, are released into the extracellular environment. Conversely to apoptosis, which is characterized by limited DMAP release, the massive cytoplasm spillover observed during NEC induces an important immune response.

Both membrane death receptors activation (via TNF-α, TRAIL, FasL) and intracellular stimulation (via genetic damage, hypoxia, osmotic stress, or starvation) trigger apoptosis [29]. The latter, known as intrinsic (e.g., mitochondrial) apoptosis, depends on mitochondria-secreted factors including cytochrome c, SMAC/DIABLO, HrtA2/Omi, and AIF that stimulate apoptosis. Intracellular cytotoxic stimuli activate BH3-only proteins leading to BAX and BAK activation, inducing the formation of mitochondrial permeability transition pores on the outer mitochondrial membrane. This feature stimulates the release of cytochrome c into the cytoplasm and its interaction with apoptotic protease activating factor 1 (APAF1) favoring the formation of the apoptosome. This molecular complex in turn stimulates procaspase 9, 3, and 7 cleavage leading to the dysfunction or disruption of cellular components and apoptosis.

The interaction of membrane death receptors and death ligands initiates extrinsic (e.g., extracellular) apoptosis. The formation of death-included signaling complex (DISC), which contains TNFR1, Fas-FasL, death receptor 4 (DR4), death receptor 3 (DR3), and tumor necrosis factor superfamily 10 (known as TRAIL/Apo2L), is the first step of the pathway [30]. The interaction of Fas with FasL induces conformational changes of the complex that favors the exposure of Fas death effector domains, facilitating the recruitment of adaptor FADD, and the activation of procaspase 8 and 10. In turn, the cleavage or activation of procaspases 8 and 10 stimulates procaspase 3 and 7 cleavage leading to the enzymatic activation of target proteins and apoptosis [30].

Similar to apoptosis, NEC is triggered by the interaction of death receptors (Fas, TNFR1, and TNFR2) with death ligands (TNF-α, FasL, and TRAIL). In apoptosis, NEC induction requires the inhibition of caspase signaling pathways (Figure 1) [22,31]. The interaction of death ligands with membrane receptors provokes conformational changes that induce the recruitment of TNF receptor-associated death domain protein (TRADD), RIP1, tumor necrosis factor-related factor 2 (TRAF2), the cellular inhibitors of apoptosis cIAP1/2 and ubiquitination complex, favoring the formation of a TNFR complex (complex I) [32]. This protein complex is responsible for cell fate, i.e., death or survival, depending on the activation of downstream pathways via ubiquitination and phosphorylation activities. Through the activation of NF-kB, complex I stimulates cell survival and promotes inflammation [33,34,35]. The ubiquitination status of RIP1 favors the recruitment of transforming growth factor β kinase 1 (TAK1), TAK1-binding protein 2 (TAB2), and TAB3 (TAK complex), and the IKK complex (IKKα and IKKβ). In this condition, RIP1 functions as a scaffold protein that favors the activation of NF-kB (e.g., cIAP2-mediated degradation of NF-kB inhibitory protein Ikβ of the IKK complex), which, following nuclear translocation, stimulates cell survival (e.g., activation of antiapoptotic genes including cFLIP) and inflammation. Apart from enzymatic activity, cFLIP is highly homologous to caspase 8, and interacting with caspase 8 inhibits its activation, favoring cell survival [36,37,38,39].

The ubiquitination status of complex I elements stabilizes its localization on the cell membrane, impeding the formation of complex II and favoring cell survival [38]. Reduced RIP1 ubiquitination favors the formation and the cytoplasmic localization of complex II (IIa, IIb, and IIc/necrosome) leading to apoptosis or NEC [37,38,39]. The formation of complex IIa, which contains procaspase 8, TRADD, and FADD, allows for caspase 8 and subsequently caspase 3 activation, leading to apoptosis. The inhibition of RIP1 polyubiquitination in complex I favors its cytoplasmic localization and the interaction with FADD and procaspase 8, leading to the formation of complex IIb (RIP1/FADD/procaspase 8). Regarding complex IIa, the activation of caspase 8 induces caspase 8-dependent apoptosis as well.

Phosphorylated RIP1 (p-RIP1) interacts with RIP3 and favors its phosphorylation (p-RIP3). Following the inhibition of caspase 8 activation (e.g., via cFLIP), the p-RIP1/p-RIP3 complex recruits MLKL favoring the formation of complex IIc/necrosome. Phosphorylation on threonine 357/serine 358 of MLKL provokes its translocation on the plasma membrane leading to the activation of sodium and calcium channels, and inducing membrane dysfunction, cell rupture, and NEC execution [40,41,42].

The levels of cFLIP influence the switch apoptosis/NEC. Elevated cFLIP expression inhibits caspase 8 by forming heteromeric complex caspase 8–cFLIP, thereby blocking apoptosis dependent on complexes IIa and IIb.

Besides plasma membrane localization, the RIP1/RIP3/MLKL complex translocates to the mitochondrial membrane provoking mitochondrial dysfunction (e.g., mitochondrial permeability transition), and inducing the production of ROS and the activation of mitochondrial phosphoglycerate mutase 5 (PGAM5). Activated PGAM5 recruits mitochondrial dynamin-related protein (DRP1) leading to mitochondrial fragmentation [43]. ROS are crucial for NEC, and some evidence links RIP3 to ROS levels. RIP3 stimulates mitochondrial enzymes, including pyruvate dehydrogenase complex, glutamine synthetase, and glutamate dehydrogenase, promoting ROS production and NEC [44]. However, the involvement of mitochondria in NEC is controversial. Indeed, cells depleted of mitochondria through forced mitophagy undergo NEC, implying that mitochondria or mitochondrial metabolism are not essential for this type of RCD [45]. This scenario reveals that the kinase-dependent NEC can be viewed as a rescue mechanism of cell death functioning when caspase-mediated apoptosis fails [26,40,46].

## 3. Necroptosis and Necroptosis Inducers in Prostate Cancer

Though reported since 2005, the induction of NEC for fighting PCa is still scarcely considered. RIP3 is decreased in PCa specimens and in cell lines (e.g., PC3, DU145, and 22Rv1). Moreover, in advanced PCa samples, RIP3 is significantly downregulated compared to normal tissue [47]. The reduced expression of RIP3 correlates with tumor size and prostate-specific antigen (PSA) levels [48]. High or normal levels of RIP3 counteract disease progression by favoring MLKL phosphorylation, leading to NEC. The overexpression of RIP3 in PC3 and 22Rv1 cell lines induces G2 cell-cycle arrest, reduces cell survival, proliferation, invasion (increased MMP2 and MMP9, vimentin, fibronectin, and N-cadherin), and favors NEC (phosphorylation of MLKL) [47]. In addition, compared to corresponding parental tumors, tumorigenesis and tumor volume are reduced in mice bearing RIP3-overexpressing PC3 and 22Rv1 tumors [47].

The seven members’ sirtuin (SIRT) family of proteins shows enzymatic activity implicated in numerous cellular functions, including DNA damage repair, senescence, metabolism, and tumor development and progression. Two enzymes of this family, SIRT3 and SIRT6, are involved in NEC induction in PCa. The increased expression of SIRT3 and SIRT6 is significantly associated with nodal metastasis and Gleason score, and negatively impacts on overall patient survival [49]. Therefore, the silencing of SIRT3 and SIRT6 in LNCaP, DU145, and PC3 cells significantly reduces cell growth, which is paralleled by RIP3-mediated NEC induction (e.g., increased phosphorylation of RIP3 and MLKL). These results were confirmed in vivo in SIRT3 and SIRT6 silenced LNCaP tumor xenografts that showed reduced tumor volume compared to the corresponding tumors expressing normal enzyme levels.

**Figure 2:**
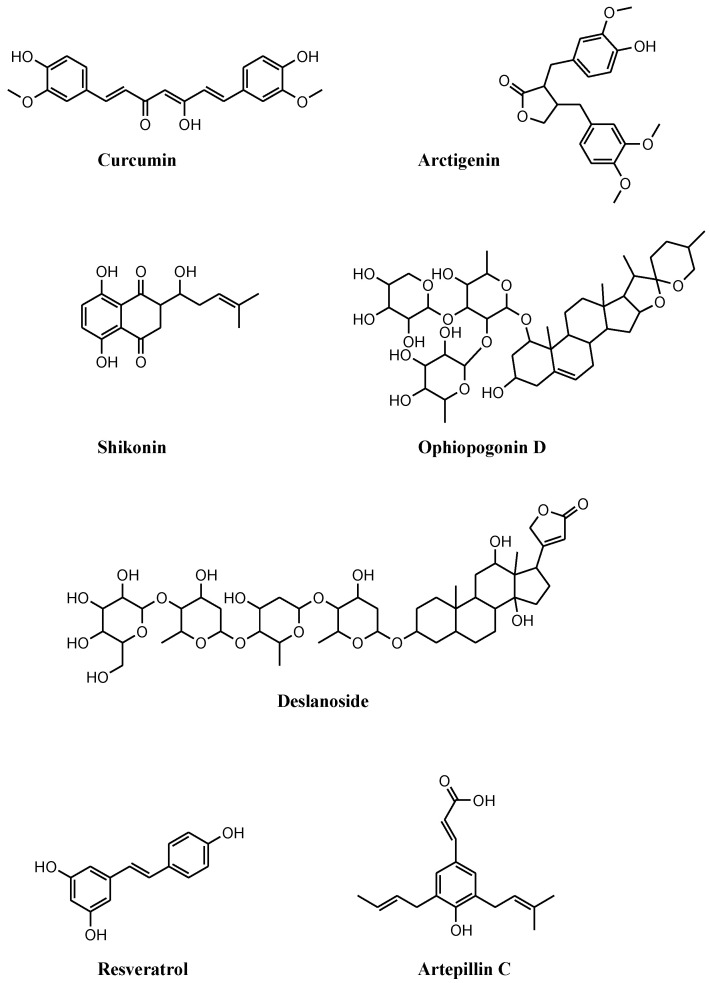
Chemical structures of compounds of natural origin that induce necroptosis in prostate cancer cell lines. These compounds stimulate necroptosis by inducing (i) the dysfunction of mitochondrial membrane potential, curcumin, and arctigenin; (ii) cell-cycle arrest, shikonin; (iii) FADD stabilization, ophiopogonin D’; (iv) modulation of multiple necroptosis pathways, deslanoside; (v) reactive oxygen species, resveratrol; (vi) RIP1/3 activation following combination with autophagy inhibitors, artepillin C.

**Figure 3:**
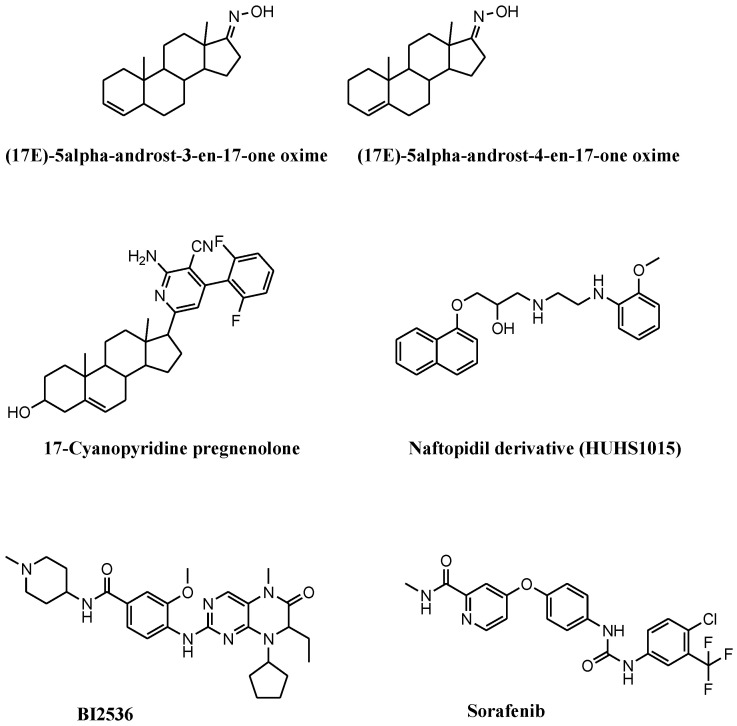
Chemical structures of synthetic and semisynthetic small molecules that induce necroptosis in prostate cancer cell lines. These compounds stimulate necroptosis by (i) inducing mitochondrial membrane permeability, (17E)-5α-androst-3-en-17-one oxime and (17E)-androst-4-en-17-one oxime; (ii) inducing reactive oxygen species and cell-cycle arrest, 17-cyanopyridine pregnenolone; (iii) inducing cell growth inhibition following combination with necrostatin, HUHS1015; (iv) inhibiting Polo-like kinase 1, BI2536; (v) inhibiting kinases, sorafenib.

Compounds that induce NEC are receiving considerable attention, and several molecules, though not specific NEC inducers, showed antitumor activity on PCa cell lines (Figure 2 and Figure 3). Available compounds are: (i) of natural origin, (ii) synthetic and semisynthetic small molecules, and (iii) selenium and selenium-based nanoparticles (SeNPs).

### 3.1. Compounds of Natural Origin

Numerous compounds of natural origin are reported to induce NEC in PCa cell lines.

Potent cytotoxic activity against DU145, PC3, and LNCaP cell lines was reported for Lu01-M, a metabolite isolated from marine actinomycetes *Streptomyces* sp. (Table 2) [50].This compound inhibits the cell proliferation and migration of PCa cell lines, stimulates G2/M cell-cycle arrest, and induces cytotoxicity via multiple mechanisms. Cells exposed to Lu01-M show endoplasmic reticulum stress (increased Grp78/Bip), apoptosis induction (PARP1 cleavage, decreased mitochondrial membrane potential, and cytochrome c release), and autophagy (increased p62 and LC3B). However, despite PC3-treated cells show NEC induction (increased TNFR2, p-RIP1 and p-MLKL, and no activation of caspase 8), cotreatment with Nec1 does not improve cell proliferation and survival.

The simultaneous induction of apoptosis and NEC was reported in curcumin-treated PC3 cells cultured under acidic conditions, acidity-tolerant PC3AcT cells (Figure 2 and Table 2) [51]. The peculiar growth conditions favor the shift from glucose to oxidative phosphorylation metabolism (Warburg phenotype) potentiating curcumin efficacy. Compared to PC3, PC3AcT cells are more sensitive to curcumin at nontoxic concentrations on normal RWPE-1 and HPrEC prostate cells. The reduced proliferation of PC3AcT cells correlates with increased ROS levels and DNA damage, the dysfunction of mitochondrial membrane potential, and ATP release, which provoke apoptosis (caspase-3 and PARP1 cleavage) and NEC (increased p-RIP3 and p-MLKL levels). Such a behavior is reversed following pretreatment with apoptosis and NEC inhibitor, or by replacing ATP and reducing ROS levels by N-acetyl cysteine (NAC).

Besides curcumin efficacy, the Warburg phenotype potentiates the antiproliferative activity of the mitochondrial complex I inhibitor arctigenin (Figure 2 and Table 2) [52]. The compound significantly reduces the growth of PC3 and PC3AcT cells with marginal toxicity on normal HPrEC and RWPE-1 cells. Acidic conditions improve arctigenin potency and docetaxel activity in drug combination studies. Besides mitochondrial dysfunctions (mitochondrial membrane depolarization and ATP depletion), arctigenin exposure increases the expression of cell communication network factor 1 (CCN1) and the levels of ROS, leading to sub-G0/G1 cell-cycle arrest, apoptosis (e.g., reduction in Bcl2, upregulation of Mcl1 and Bax, and caspase 3 activation), and NEC (increased p-RIP3 and p-MLKL expression). These findings are corroborated by siRNA-mediated CCN1 silencing experiments and by the rescue of cell viability observed following pretreatment with NAC or Nec1 before arctigenin exposure.

Shikonin (SHI) is a drug of traditional Chinese medicine showing interesting antiproliferative activity on a panel of PCa cell lines that are sensitive (PC3, DU145, LNCaP, and 22Rv1) and resistant to docetaxel (PC3-res, DU145-res, LNCaP-res, and 22Rv1-res) (Figure 2 and Table 2) [53]. Reduced cell growth observed following exposure to SHI matches with cell-cycle arrest in G2/M and S. Cell-cycle perturbations parallel with the modulation of cyclin A and B, p21, p27, and CDK1 (reflecting G2/M arrest), and of cyclin A and CDK2 (supporting S phase block) expression levels. Exposure to SHI triggers apoptosis and NEC in both parental and docetaxel-resistant PCa cells. The reduction in caspase 8 activation coupled with increased p-RIP1 and p-RIP3 expression, more evident in aggressive androgen-insensitive PC3 and DU145 cells, accounts for NEC as a dominant mechanism of cell death. This finding is corroborated by the observation that NEC induction is blocked by exposure to Nec1.

Natural compound ophiopogonin D’ (OPD, Figure 2 and Table 2) extracted from *Ophiopogon japonicus*, exhibits antiproliferative activity against PCa cells. OPD induces NEC or apoptosis depending on the cellular context [48]. NEC induction (increased expression of RIP1, MLKL, and RIP3) is observed in androgen-sensitive LNCaP cells exposed to OPD. This behavior is counteracted by the combined exposure to OPD and NEC inhibitors necrosulfonamide and Nec1. Apoptosis is observed in OPD-treated PC3 cells. The different cell response depends on FADD levels. FADD, caspase 8, and RIP1 form a death-inducing signalling complex that potentially induces both apoptosis and NEC depending on FADD levels. In PC3 cells, OPD reduces FADD expression leading to apoptosis via ROS production. In LNCaP cells, OPD increases FADD levels, which stabilize the death-inducing signaling complex promoting NEC.

NEC was reported by Rizzi et al. in PCa cell lines exposed to green tea extract (Polyphenon E) (Table 2) [54]. The different cell response of PNT1a and PC3 cells following treatment with Polyphenon E indicates that the tumor stage of PCa from which cell lines were derived (initial for PNAT1a, and advanced for PC3 cells) drives cellular behavior. PNT1a cells are 4 times more sensitive than PC3 cells are. Different cell-cycle perturbations are observed in PNT1a (G0/G1 arrest) compared to PC3 (G2/M block) cells. Endoplasmic reticulum stress and the increment of unfolded proteins stimulate protective autophagy in PNT1a cells, leading to a survival response. The more important and persistent alterations of the endoplasmic reticulum, coupled with increased unfolded proteins observed in Polyphenon E-treated PC3 cells, provoke the upregulation of GADD153/CHOP level, which in turn activates PUMA, leading to NEC. Although typical markers of NEC are not evaluated, peculiar intracellular morphological changes observed for PC3 cells account for NEC.

Anticancer effects of cardiac glycoside deslanoside, which is found in the leaves of *Digitalis lanata*, were reported in PCa cell lines (Figure 2 and Table 2) [55]. This drug shows in vitro antiproliferative activity and tumor growth inhibition in vivo on 22Rv1, PC3, and DU145 cells. Deslanoside inhibits tumor growth by reducing cell proliferation (reduced Ki67 index), stimulating G2/M cell-cycle arrest (increased p21, cyclin D1, CDK1, and cyclin B1 expression), and inducing apoptosis (caspase 3 and 9, and PARP1 cleavage). Moreover, reduced cell migration and invasion capabilities were reported in deslanoside-exposed PCa cells. The antitumor activity of deslanoside obviously does not rely on its action on the Na^+^/K^+^-ATPase, but it depends on the modulation of multiple pathways, including that implicated in NEC, as observed in the genomewide expression profiling of 22Rv1 and PC3 cell lines.

The antitumor activity of resveratrol was studied in several tumors, including PCa [56]. Resveratrol improves docetaxel potency in PCa cell lines (Figure 2 and Table 2). NEC induced by docetaxel in LNCaP cells is potentiated by a combination with resveratrol [57]. Compared to single-drug exposure, the drug combination synergistically reduces cell growth, induces G2/M cell-cycle arrest, and increases ROS production, which lead to DNA damage (activation of ATM and ATR kinases) and mitochondrial dysfunction (loss of membrane potential), apoptosis induction (reduced Bcl2 and increased Bax levels), and NEC (increased p-RIP3 and p-MLKL). Since NAC counteracts apoptosis and NEC, ROS are recognized as upstream molecules inducing both types of RCD.

Cinnamic acid derivative artepillin C (ArtC) contained in the extracts of Brazilian green propolis possesses anticancer activity against PCa cell lines (Figure 2 and Table 2) [58]. Endo and coworkers demonstrated that CWR22Rv1 cells treated with ArtC activate apoptosis (caspase-3 and PARP1 cleavage) and protective autophagy (increased LC3B expression). Autophagy opposes apoptosis and favors cell survival. Combined exposure to ArtC and autophagy inhibitors (wortmannin, chloroquine and U0126) potentiates apoptosis and induces NEC (increased p-RIP1/3).

### 3.2. Synthetic and Semisynthetic Small Molecules

Gomes and coworkers combined steroidal structure and oximes moieties, two chemical features with proven anticancer activity, for the synthesis of a series of steroid-oxime derivatives (Figure 3 and Table 2) [59]. Two of these derivatives, (17E)-5α-androst-3-en-17-one oxime (3,4-OLOX) and (17E)-androst-4-en-17-one oxime (4,5-OLOX), showed antiproliferative activity on a panel of tumor cell lines, including PCa cells. The reduced proliferation observed in 3,4-OLOX- and 4,5-OLOX-treated PC3 cells depends on apoptosis (reduced Bcl2 and increased Bax) and NEC (decreased mitochondrial membrane potential) induction. This behavior, which is associated with increased ROS production, was not observed in healthy colon CCD841 CoN cell line. Apoptosis and NEC occur simultaneously, while NEC subsequently dominates. The shift from apoptosis to NEC depends on ROS accumulation into the mitochondria and on alterations of mitochondrial membrane permeability. The compounds show hemocompatibility (no alterations of the red blood cells) and safety for intravenous administration.

The 17-cyanopyridine derivatives of pregnenolone are steroid structures showing interesting cytotoxicity on PCa cell lines (Figure 3 and Table 2). Among these derivatives, difluorinated 2-amino-4-aryl-3-cyanopyridine in position 17 of ring D of pregnenolone is the most active on PC3 cells [60]. Reduced proliferation and cell migration was observed in PC3 cells exposed to the compound. These features are in parallel with increased ROS levels, G0/G1 cell-cycle arrest, modulation of epithelial to mesenchymal transition markers (e.g., reduced N-cadherin and vimentin, and increased E-cadherin) and the upregulation of NEC-associated proteins, including p-RIP1/3 and p-MLKL, which account for the involvement of NEC as a mechanism of the antitumor activity of the compound.

Naftopidil analog 1-[2-(2-methoxyphenylamino) ethylamino]-3-(naphthalene-1-yloxy) propan-2-ol (HUHS1015) induces NEC in PC3, DU145, and LNCaP cell lines (Figure 3 and Table 2) [61]. HUHS1015-treated PCa cells showed reduced proliferation. Despite the markers of NEC not being evaluated in this study, NEC is proposed as the involved cell death mechanism on the basis of the reduced cell death observed with combining Nec1 with HUHS1015. HUHS1015 demonstrated antitumor activity in vivo in (NCI-H2052), gastric (MKN45), and colorectal (CW2) tumor models. No in vivo data are available for PCa.

The link between NEC and mitotic kinase Polo-like kinase 1 (Plk1) was reported by Deeraksa et al. in LNCaP cells (Figure 3 and Table 2) [62]. Compared to parental androgen-sensitive LNCaP cells, androgen-insensitive reprogrammed LNCaP-AI cells upregulate Plk1, which regulates cell growth. In androgen deprivation, LNCaP-AI cells show dependency on Plk1 for growth and consequent sensitivity to Plk1 inhibitor BI2536. LNCaP-AI cells treated with BI2536 increase PARP1 cleavage with no activation of caspases or the increment of apoptotic markers. BI2536-treated cells are giants, multinuclear, and invaded by nuclear vesicles, which are typical morphological alterations of NEC. These features reverse following cotreatment with an NEC inhibitor. No upregulation of Plk1 and negligible response to BI2536 has been reported for parental LNCaP cells.

Another interesting drug inducing NEC in PCa cell lines is second-generation tyrosine kinase inhibitor sorafenib (Figure 3 and Table 2). This drug impairs the functions of several kinases (e.g., PDGFR-β, cKIT, Raf1, VEGFR-2 and 3, and Flt3) and induces NEC in Atg5-defective DU145 cells. Sorafenib exposure provokes mitochondrial alterations and autophagy in DU145 cells, which are counteracted by the silencing of ULK1 or Beclin1. Despite LC3B being defective, an autophagosome containing p62 and RIP1 is formed in sorafenib-exposed DU145 cells. This observation is corroborated by the finding that Nec1 counteracts the activation of the RIP1/RIP3/MLKL pathway, thus attenuating NEC induction [63]. In this context, the absence of Atg favors the p62-mediated recruitment of RIP1 into the autophagosome, switching the induction of autophagy towards NEC.

### 3.3. Selenium and Selenium-Based Nanoparticles

Selenium levels show inverse correlation with PCa risk and mortality. Moreover, the association between selenium and cancer prevention or treatment response of PCa is demonstrated in large prospective clinical trials [64]. An interesting strategy for fighting cancer is the administration of selenium and its delivery through SeNPs (Table 2) [65,66,67]. Depending on the cellular context, selenite, an inorganic form of selenium, activates apoptosis or NEC. Selenite induces apoptosis in androgen-sensitive LNCaP cells, and NEC in PC3 cells [64]. The different cell response depends on p53 status. ROS induced by selenite activates wt p53 in LNCaP cells leading to apoptosis. p53-deficient PC3 and p53-mutated DU145 cells undergo NEC depending on intracellular ATP levels. Indeed, the enhancement of ROS levels in selenite-treated PC3 and DU145 cells impairs phosphofructokinase activity, leading to ATP depletion and favoring NEC. This finding is confirmed by the lack of activation of caspases and PARP1, and by the unchanged Bax expression following selenite exposure. On the other hand, RIP3 inhibitor dabrafenib and NAC protect PC3 and DU145 cells from selenite-induced NEC.

Sonkusre and coworkers reported the production of biogenic SeNPs from *Bacillus licheniformis* endowed with NEC activity in LNCaP and PC3 cells [66,67]. NEC induced by SeNPs depends on ROS elevation, and is corroborated by the increased expression of TNF-α, interferon regulatory factor 1 (IRF1), and RIP1. Since no variations in p-RIP3 and p-MLKL were observed, NEC is supposed to occur through the activation of a nonconventional NEC pathway. This finding is consistent with the lack of expression of RIP3 in PC3 cells due to gene methylation. However, cotreatment with Nec1 improves SeNPs-treated PC3 cell viability, thus supporting the induction of NEC as the cell death mechanism.

## 4. Ongoing Clinical Trials Containing Necroptosis Inducers in Prostate Cancer

Some compounds reported in this review are contained in clinical trials enrolling patients suffering from PCa (Table 3, www.clinicaltrials.gov, accessed on 23 February 2022).

Curcumin alone (NCT03769766, phase III; NCT03211104; NCT02064673, phase III) or in combination with piperine (NCT04731844, phase II), ursolic acid (NCT04403568, phase I), vitamin D, omega 3 and turmeric (NCT03290417), taxotere (NCT02095717, phase II) and radiotherapy (NCT01917890; NCT02724618, phase II; NCT03493997, phase II) is studied in PCa patients. Despite studies implying the measure of different biomarkers, mostly PSA, no specific markers of NEC are considered. Only trials NCT04403568 and NCT01917890 contemplate the evaluation of TNF-α and NF-kB. These two molecules are involved in NEC, though they are not specific biomarkers.

Polyphenon E was included in numerous studies as supplementary diet in subjects at high risk of developing PCa (prostatic hyperplasia) or in postsurgery patients (NCT00596011, phase II; NCT00676780, phase II; NCT01340599, phase II; NCT00459407, phase I; NCT00253643; NCT04597359, phase II). Polyphenon E-based studies contemplated the measure of numerous biological markers (e.g., PSA, Ki67, Bcl2, Cyclin D, p27, VEGF, CD31, MMP2 and 9, IGF1, HGF, FASN) but not specifically related to NEC.

Sorafenib alone (NCT00090545, phase II; NCT00694291, phase II; NCT00466752, phase I; NCT00093457, phase II) or in combination with leuprolide acetate or bicalutamide (NCT00924807, phase I-II), docetaxel (NCT00589420, phase II; NCT00619996, phase II), taxotere (NCT00405210, phase I) mitoxantrone (NCT00452387, phase II) or gleevec (NCT00424385, phase I) is investigated in PCa patients. In all these studies, PSA is used as biological marker. The trial NCT00619996 contemplates the measure of p-ERK and VEGF-R2 as well. Interestingly, in trial NCT00466752, samples from PCa patients collected before and after sorafenib administration were analyzed for gene and protein expression by microarray, Western blot, and immunohistochemistry. In this context, the levels of p-ERK, p-AKT, p-S6- kinase and caspase-3 expression, and the Ki67 index were considered. Though the study is completed, results are not yet available. In sorafenib-containing clinical trials, specific biomarkers of NEC are also not measured.

Selenite in combination with docetaxel (NCT01155791, phase I) or with radiotherapy (NCT02184533, phase I) is studied in PCa-affected patients. In these studies, PSA was the only considered biomarker.

## 5. Conclusions

The scarce response of CRPC to available therapies, including latest-generation drugs, is still an urgent problem, and the discovery of new effective compounds on this disease is mandatory. In this context, the study of cellular mechanisms of NEC and the development of compounds that induce such a type of RCD is opening a new scenario. Although NEC is a recently discovered RCD, several molecules that induce NEC are already available. In spite of numerous in vitro investigations performed on cell lines recapitulating the wide range of PCa features, the relation between AR status and drug response is still not clarified. The study of inflammatory reactions induced by NEC, which depend on the release of proinflammatory molecules, requires deep investigation. Moreover, specific markers of NEC, e.g., p-RIP1, p-RIP3 and p-MLKL, are not evaluated in all studies, and diverse in vitro investigations inferred the induction of NEC by cell morphological changes or drug combination studies with NEC inhibitors (e.g., studies with Polyphenon E, steroid-oxime derivatives, naftopidil derivatives, BI2536, selenite, and SeNPs). Only deslanoside was tested in vivo on PCa models and, in spite of numerous ongoing clinical trials containing NEC inducing compounds, only few enrolled patients suffering from PCa. However, none of these trials contemplates the measure of specific biomarkers of NEC.

Despite NEC and apoptosis share some properties and effector molecules, the molecular mechanisms governing the switch of apoptosis–NEC are not fully understood. The elucidation of these mechanisms is expected to solicit the discovery of new and more specific compounds that are capable of inducing NEC cell death in tumors resistant to apoptosis.

In conclusion, the induction of NEC as a strategy for fighting PCa is still in its infancy. The implementation of preclinical and clinical investigations in this field is expected to improve the therapies for CRPC.

## Figures and Tables

**Table 1:** Comparison of necroptosis-, necrosis-, and apoptosis-regulated cell death properties.

	Characteristic	Necroptosis	Necrosis	Apoptosis
Involved proteins	RIP3	+	/	/
	MLKL	+	/	/
	Caspase 3	/	/	+
Cell properties	Membrane perforation	+	+	/
	Membrane blebbing	/	/	+
	DNA fragmentation	+	+	+
	Cell lysis and swelling	+	+	/
	Inflammation	+	+	/

**Table 2:** Compounds that induce necroptosis in prostate cancer cell lines.

Class of Compounds	Compound	Cell Lines	In Vivo Studies	Necroptosis Inhibitors	Necroptosis Markers
Compounds of natural origin	Lu01-M	DU145, PC3, LNCaP	No	Yes	TNFR2, p-RIP1, p-MLKL
	Curcumin	PC3, PC3AcT	No	Yes	p-RIP3, p-MLKL
	Arctigenin	PC3, PC3AcT	No	Yes	p-RIP3, p-MLKL
	Shikonin	Pc3, DU145, LNCaP, 22Rv1	No	Yes	p-RIP1, p-RIP3
	Ophiopogonin D’	PC3, LNCaP	No	Yes	p-RIP1, p-RIP3, p-MLKL
	Polyphenon E	PC3, PNT1a	No	No	Cell morphological changes
	Deslanoside	PC3, DU145, 22Rv1	PC3, 22Rv1	No	Genome wide expression profile
	Resveratrol	LNCaP	No	No	p-RIP3, p-MLKL
	Artepillin C	CW22Rv1	No	No	p-RIP3
Synthetic and semisynthetic small molecules	Steroid-oxime derivatives	PC3	No	No	Decreased mitochondrial membrane potential
	17-cyanopyridine pregnenolone derivative	PC3	No	No	p-RIP1, p-RIP3, p-MLKL
	Naftopidil derivative (HUHS1015)	PC3, DU145, LNCaP	No ^a^	yes	In vitro drug combination studies with necrostatin 1
	BI2536	LNCaP, LNCaP-AI	No	Yes	In vitro drug combination studies with necrostatin 1
	Sorafenib	DU145	No	Yes	p-RIP1, p-RIP3, p-MLKL
Selenium and selenium-based nanoparticles	Selenite	PC3, DU145, LNCaP	No	Yes	In vitro drug combination studies with dabrafenib and N-acetyl cysteine
	Selenium-based nanoparticles	PC3, LNCaP	No	Yes	In vitro drug combination studies with necrostatin 1

^a^ HUHS1015 was tested in vivo in mesothelioma NCI-H2052, gastric MKN45, and colorectal CW2 tumors.

**Table 3:** Clinical trials containing necroptosis inducers ongoing in prostate cancer ^a^.

Compound	NCT Number	Markers	Phase
Curcumin	NCT03769766	PSA	III
	NCT03211104	PSA	na
	NCT02064673	PSA	III
Curcumin and piperine	NCT04731844	nd	II
Curcumin and ursolic acid	NCT04403568	p65, NF-kB	I
Curcumin and Vitamin D, omega 3, turmeric	NCT03290417	PSA	na
Curcumin and taxotere	NCT02095717	PSA	III
Curcumin and radiotherapy	NCT01917890	TNF-α, NF-kB	na
	NCT02724618	PSA	II
	NCT03493997	nd	II
Polyphenon E	NCT00596011	PSA	II
	NCT00676780	PSA, VEGF, HGF	II
	NCT01340599	PSA, Ki67, Bcl2, Cyclin D, p27, VEGF, CD31, MMP2 and 9, IGF1	II
	NCT00459407	MMP2, MMP9, IGF1	I
	NCT00253643	FASN, Ki67	na
	NCT04597359	PSA, Ki67	II
BI2536	NCT00706498	PSA	II
Sorafenib	NCT00090545	PSA	II
	NCT00694291	PSA	II
	NCT00466752	PSA, p-ERK, p-AKT, p-S6-kinase, caspase 3, Ki67	I
	NCT00093457	PSA	II
Sorafenib and leuprolide or bicalutamide	NCT00924807	PSA	I-II
Sorafenib and docetaxel	NCT00589420	PSA	II
	NCT00619996	PSA, p-ERK, VEGF-R2	II
Sorafenib and taxotere	NCT00405210	PSA	I
Sorafenib and mitoxantrone	NCT00452387	PSA	II
Sorafenib and gleevec	NCT00424385	PSA	I
Selenite and docetaxel	NCT01155791	PSA	I
Selenite and radiotherapy	NCT02184533	PSA	I

^a^ Clinical data are from ClinicalTrials.gov, a service of the U.S. National Institutes of Health. www.clinicaltrial.gov (accessed on 23 February 2022). nd, not defined; na, not available.

## References

[1] Tang D., Kang R., Berghe T.V., Vandenabeele P., Kroemer G. (2019). The molecular machinery of regulated cell death. Cell Res..

[2] Choi J.J., Reich C.F., Pisetsky D.S. (2004). Release of DNA from dead and dying lymphocyte and monocyte cell lines in vitro. Scand. J. Immunol..

[3] Kang R., Zeng L., Zhu S., Xie Y., Liu J., Wen Q., Cao L., Xie M., Ran Q., Kroemer G. (2018). Lipid peroxidation drives gasdermin D-mediated pyroptosis in lethal polymicrobial sepsis. Cell Host. Microbe..

[4] Canli Ö., Alankuş Y.B., Grootjans S., Vegi N., Hültner L., Hoppe P.S., Schroeder T., Vandenabeele P., Bornkamm G.W., Greten F.R. (2016). Glutathione peroxidase 4 prevents necroptosis in mouse erythroid precursors. Blood J. Am. Soc. Hematol..

[5] Haberzettl P., Hill B.G. (2013). Oxidized lipids activate autophagy in a JNK-dependent manner by stimulating the endoplasmic reticulum stress response. Redox Biol..

[6] Wang Y., Wu N., Jiang N. (2021). Autophagy provides a conceptual therapeutic framework for bone metastasis from prostate cancer. Cell Death Dis..

[7] Zaffaroni N., Beretta G.L. (2021). Nanoparticles for Ferroptosis Therapy in Cancer. Pharmaceutics.

[8] Zaffaroni N., Beretta G.L. (2022). Ferroptosis inducers for prostate cancer therapy. Curr. Med. Chem..

[9] Goodall M.L., Cramer S.D., Thorburn A. (2016). Autophagy RIPs into cell death. Cell Cycle.

[10] Karki R., Sharma B.R., Lee E., Banoth B., Malireddi R.K.S., Samir P., Tuladhar S., Mummareddy H., Burton A.R., Vogel P. (2020). Interferon regulatory factor 1 regulates PANoptosis to prevent colorectal cancer. JCI Insight.

[11] Place D.E., Lee S., Kanneganti T.D. (2021). PANoptosis in microbial infection. Curr. Opin. Microbiol..

[12] Heidaryan F., Bamehr H., Babaabasi B., Emamvirdizadeh A., Mohammadzadeh N., Khalili A. (2020). The Trend of ripk1/ripk3 and mlkl Mediated Necroptosis Pathway in Patients with Different Stages of Prostate Cancer as Promising Progression Biomarkers. Clin. Lab..

[13] Liu W., Jin W., Zhu S., Chen Y., Liu B. (2022). Targeting regulated cell death (RCD) with small-molecule compounds in cancer therapy: A revisited review of apoptosis, autophagy-dependent cell death and necroptosis. Drug Discov. Today.

[14] Carneiro B.A., El-Deiry W.S. (2020). Targeting apoptosis in cancer therapy. Nat. Rev. Clin. Oncol..

[15] Fulda S., Vucic D. (2012). Targeting IAP proteins for therapeutic intervention in cancer. Nat. Rev. Drug Discov..

[16] Yan J., Wan P., Choksi S., Liu Z.G. (2022). Necroptosis and tumor progression. Trends Cancer.

[17] Nie Z., Chen M., Gao Y., Huang D., Cao H., Peng Y., Guo N., Zhang S. (2021). Regulated Cell Death in Urinary Malignancies. Front. Cell Dev. Biol..

[18] Mohammad R.M., Muqbil I., Lowe L., Yedjou C., Hsu H.Y., Lin L.T., Siegelin M.D., Fimognari C., Kumar N.B., Dou Q.P. (2015). Broad targeting of resistance to apoptosis in cancer. Semin. Cancer Biol..

[19] Sung H., Ferlay J., Siegel R.L., Laversanne M., Soerjomataram I., Jemal A., Bray F. (2021). Global Cancer Statistics 2020: GLOBOCAN Estimates of Incidence and Mortality Worldwide for 36 Cancers in 185 Countries. CA Cancer J. Clin..

[20] Nuhn P., De Bono J.S., Fizazi K., Freedland S.J., Grilli M., Kantoff P.W., Sonpavde G., Sternberg C.N., Yegnasubramanian S., Antonarakis E.S. (2019). Update on systemic prostate cancer therapies: Management of metastatic castration resistant prostate cancer in the era of precision oncology. Eur. Urol..

[21] Martin-Sanchez D., Fontecha-Barriuso M., Sanchez-Niño M.D., Ramos A.M., Cabello R., Gonzalez-Enguita C., Linkermann A., Sanz A.B., Ortiz A. (2018). Cell death-based approaches in treatment of the urinary tract-associated diseases: A fight for survival in the killing fields. Cell Death Dis..

[22] Degterev A., Huang Z., Boyce M., Li Y., Jagtap P., Mizushima N., Cuny G.D., Mitchison T.J., Moskowitz M.A., Yuan J. (2005). Chemical inhibitor of nonapoptotic cell death with therapeutic potential for ischemic brain injury. Nat. Chem. Biol..

[23] Galluzzi L., Vitale I., Aaronson S.A., Abrams J.M., Adam D., Agostinis P., Alnemri E.S., Altucci L., Amelio I., Andrews D.W. (2018). Molecular mechanisms of cell death: Recommendations of the Nomenclature Committee on Cell Death 2018. Cell Death Differ..

[24] Liu X., Xie X., Ren Y., Shao Z., Zhang N., Li L., Ding X., Zhang L. (2021). The role of necroptosis in disease and treatment. MedComm.

[25] Davidovich P., Kearney C.J., Martin S.J. (2014). Inflammatory outcomes of apoptosis, necrosis and necroptosis. Biol. Chem..

[26] Vanden Berghe T., Linkermann A., Jouan-Lanhouet S., Walczak H., Vandenabeele P. (2014). Regulated necrosis: The expanding network of non-apoptotic cell death pathways. Nat. Rev. Mol. Cell Biol..

[27] Laster S.M., Wood J.G., Gooding L.R. (1988). Tumor necrosis factor can induce both apoptic and necrotic forms of cell lysis. J. Immunol..

[28] Vercammen D., Brouckaert G., Denecker G., Van de Craen M., Declercq W., Fiers W., Vandenabeele P. (1998). Dual signaling of the Fas receptor: Initiation of both apoptotic and necrotic cell death pathways. J. Exp. Med..

[29] Mishra A.P., Salehi B., Sharifi-Rad M., Pezzani R., Kobarfard F., Sharifi-Rad J., Nigam M. (2018). Programmed Cell Death, from a Cancer Perspective: An Overview. Mol. Diagn. Ther..

[30] D’Arcy M.S. (2019). Cell death: A review of the major forms of apoptosis, necrosis and autophagy. Cell Biol. Int..

[31] Holler N., Zaru R., Micheau O., Thome M., Attinger A., Valitutti S., Bodmer J.L., Schneider P., Seed B., Tschopp J. (2000). Fas triggers an alternative, caspase-8-independent cell death pathway using the kinase RIP as effector molecule. Nat. Immunol..

[32] Galluzzi L., Kepp O., Chan F.K., Kroemer G. (2017). Necroptosis: Mechanisms and Relevance to Disease. Annu. Rev. Pathol..

[33] Dondelinger Y., Darding M., Bertrand M.J., Walczak H. (2016). Poly-ubiquitination in TNFR1-mediated necroptosis. Cell Mol. Life Sci..

[34] Tortola L., Nitsch R., Bertrand M.J.M., Kogler M., Redouane Y., Kozieradzki I., Uribesalgo I., Fennell L.M., Daugaard M., Klug H. (2016). The Tumor Suppressor Hace1 Is a Critical Regulator of TNFR1-Mediated Cell Fate. Cell Rep..

[35] Zhang K.X., Firus J., Prieur B., Jia W., Rennie P.S. (2011). To die or to survive, a fatal question for the destiny of prostate cancer cells after androgen deprivation therapy. Cancers.

[36] Chan F.K., Luz N.F., Moriwaki K. (2015). Programmed necrosis in the cross talk of cell death and inflammation. Annu. Rev. Immunol..

[37] Ting A.T., Bertrand M.J.M. (2016). More to Life than NF-κB in TNFR1 Signaling. Trends Immunol..

[38] Dillon C.P., Oberst A., Weinlich R., Janke L.J., Kang T.B., Ben-Moshe T., Mak T.W., Wallach D., Green D.R. (2012). Survival function of the FADD-CASPASE-8-cFLIP(L) complex. Cell Rep..

[39] Dondelinger Y., Declercq W., Montessuit S., Roelandt R., Goncalves A., Bruggeman I., Hulpiau P., Weber K., Sehon C.A., Marquis R.W. (2014). MLKL compromises plasma membrane integrity by binding to phosphatidylinositol phosphates. Cell Rep..

[40] Quarato G., Guy C.S., Grace C.R., Llambi F., Nourse A., Rodriguez D.A., Wakefield R., Frase S., Moldoveanu T., Green D.R. (2016). Sequential Engagement of Distinct MLKL Phosphatidylinositol-Binding Sites Executes Necroptosis. Mol. Cell.

[41] Cho Y.S., Challa S., Moquin D., Genga R., Ray T.D., Guildford M., Chan F.K. (2009). Phosphorylation-driven assembly of the RIP1-RIP3 complex regulates programmed necrosis and virus-induced inflammation. Cell.

[42] Sun L., Wang H., Wang Z., He S., Chen S., Liao D., Wang L., Yan J., Liu W., Lei X. (2012). Mixed lineage kinase domain-like protein mediates necrosis signaling downstream of RIP3 kinase. Cell.

[43] Wang Z., Jiang H., Chen S., Du F., Wang X. (2012). The mitochondrial phosphatase PGAM5 functions at the convergence point of multiple necrotic death pathways. Cell.

[44] Yang Z., Wang Y., Zhang Y., He X., Zhong C.Q., Ni H., Chen X., Liang Y., Wu J., Zhao S. (2018). RIP3 targets pyruvate dehydrogenase complex to increase aerobic respiration in TNF-induced necroptosis. Nat. Cell Biol..

[45] Tait S.W., Oberst A., Quarato G., Milasta S., Haller M., Wang R., Karvela M., Ichim G., Yatim N., Albert M.L. (2013). Widespread mitochondrial depletion via mitophagy does not compromise necroptosis. Cell Rep..

[46] Zhou W., Yuan J. (2014). Necroptosis in health and diseases. Semin. Cell Dev. Biol..

[47] Wang K.J., Wang K.Y., Zhang H.Z., Meng X.Y., Chen J.F., Wang P., Jiang J.H., Ma Q. (2020). Up-Regulation of RIP3 Alleviates Prostate Cancer Progression by Activation of RIP3/MLKL Signaling Pathway and Induction of Necroptosis. Front. Oncol..

[48] Lu Z., Wu C., Zhu M., Song W., Wang H., Wang J., Guo J., Li N., Liu J., Li Y. (2020). Ophiopogonin D' induces RIPK1-dependent necroptosis in androgen-dependent LNCaP prostate cancer cells. Int J. Oncol..

[49] Fu W., Li H., Fu H., Zhao S., Shi W., Sun M., Li Y. (2020). The SIRT3 and SIRT6 Promote Prostate Cancer Progression by Inhibiting Necroptosis-Mediated Innate Immune Response. J. Immunol. Res..

[50] Lin H.Y., Lin Y.S., Shih S.P., Lee S.B., El-Shazly M., Chang K.M., Yang Y.S.H., Lee Y.L., Lu M.C. (2021). The Anti-Proliferative Activity of Secondary Metabolite from the Marine Streptomyces sp. against Prostate Cancer Cells. Life.

[51] Lee Y.J., Park K.S., Lee S.H. (2021). Curcumin Targets Both Apoptosis and Necroptosis in Acidity-Tolerant Prostate Carcinoma Cells. Biomed. Res. Int..

[52] Lee Y.J., Nam H.S., Cho M.K., Lee S.H. (2020). Arctigenin induces necroptosis through mitochondrial dysfunction with CCN1 upregulation in prostate cancer cells under lactic acidosis. Mol. Cell Biochem..

[53] Markowitsch S.D., Juetter K.M., Schupp P., Hauschulte K., Vakhrusheva O., Slade K.S., Thomas A., Tsaur I., Cinatl J., Michaelis M. (2021). Shikonin Reduces Growth of Docetaxel-Resistant Prostate Cancer Cells Mainly through Necroptosis. Cancers.

[54] Rizzi F., Naponelli V., Silva A., Modernelli A., Ramazzina I., Bonacini M., Tardito S., Gatti R., Uggeri J., Bettuzzi S. (2014). Polyphenon E(R), a standardized green tea extract, induces endoplasmic reticulum stress, leading to death of immortalized PNT1a cells by anoikis and tumorigenic PC3 by necroptosis. Carcinogenesis.

[55] Liu M., Huang Q.A.J., Li L., Li X., Zhang Z., Dong J.T. (2021). The Cardiac Glycoside Deslanoside Exerts Anticancer Activity in Prostate Cancer Cells by Modulating Multiple Signaling Pathways. Cancers.

[56] Zaffaroni N., Beretta G.L. (2021). Resveratrol and Prostate Cancer: The Power of Phytochemicals. Curr. Med. Chem..

[57] Lee S.H., Lee Y.J. (2021). Synergistic anticancer activity of resveratrol in combination with docetaxel in prostate carcinoma cells. Nutr. Res. Pract..

[58] Endo S., Hoshi M., Matsunaga T., Inoue T., Ichihara K., Ikari A. (2018). Autophagy inhibition enhances anticancer efficacy of artepillin C, a cinnamic acid derivative in Brazilian green propolis. Biochem. Biophys. Res. Commun..

[59] Gomes A.R., Pires A.S., Abrantes A.M., Gonçalves A.C., Costa S.C., Varela C.L., Silva E.T., Botelho M.F., Roleira F.M.F. (2021). Design, synthesis, and antitumor activity evaluation of steroidal oximes. Bioorg. Med. Chem..

[60] Sun Y., Gao P., Zhu L., Li Z., Zhao R., Li C., Shan L. (2021). Synthesis and biological evaluation of 17-cyanopyridine derivatives of pregnenolone as potential anti-prostate cancer agents. Steroids.

[61] Nishizaki T., Kanno T., Tsuchiya A., Kaku Y., Shimizu T., Tanaka A. (2014). 1-[2-(2-Methoxyphenylamino)ethylamino]-3-(naphthalene-1- yloxy)propan-2-ol may be a promising anticancer drug. Molecules.

[62] Deeraksa A., Pan J., Sha Y., Liu X.D., Eissa N.T., Lin S.H., Yu-Lee L.Y. (2013). Plk1 is upregulated in androgen-insensitive prostate cancer cells and its inhibition leads to necroptosis. Oncogene.

[63] Kharaziha P., Chioureas D., Baltatzis G., Fonseca P., Rodriguez P., Gogvadze V., Lennartsson L., Björklund A.C., Zhivotovsky B., Grandér D. (2015). Oncotarget. 2015 Sorafenib-induced defective autophagy promotes cell death by necroptosis. Oncotarget.

[64] Cui J., Yan M., Liu X., Yin S., Lu S., Fan L., Hu H. (2019). Inorganic Selenium Induces Nonapoptotic Programmed Cell Death in PC-3 Prostate Cancer Cells Associated with Inhibition of Glycolysis. J. Agric. Food Chem..

[65] Martínez-Esquivias F., Gutiérrez-Angulo M., Pérez-Larios A., Sánchez-Burgos J., Becerra-Ruiz J., Guzmán-Flores J.M. (2021). Anticancer Activity of Selenium Nanoparticles In Vitro Studies. Anticancer Agents Med. Chem..

[66] Sonkusre P. (2020). Specificity of Biogenic Selenium Nanoparticles for Prostate Cancer Therapy With Reduced Risk of Toxicity: An in vitro and in vivo Study. Front. Oncol.

[67] Sonkusre P., Cameotra S.S.J. (2017). Biogenic selenium nanoparticles induce ROS-mediated necroptosis in PC-3 cancer cells through TNF activation. Nanobiotechnology.

